# Integrative network-based analysis on multiple Gene Expression Omnibus datasets identifies novel immune molecular markers implicated in non-alcoholic steatohepatitis

**DOI:** 10.3389/fendo.2023.1115890

**Published:** 2023-03-16

**Authors:** Jun-jie Zhang, Yan Shen, Xiao-yuan Chen, Man-lei Jiang, Feng-hua Yuan, Shui-lian Xie, Jie Zhang, Fei Xu

**Affiliations:** ^1^ Center for Molecular Pathology, Department of Basic Medicine, Gannan Medical University, Ganzhou, China; ^2^ Department of Publication Health and Health Management, Gannan Medical University, Ganzhou, China; ^3^ Department of Hepatology, The Affiliated Fifth People’s Hospital of Ganzhou, Gannan Medical University, Ganzhou, China

**Keywords:** non-alcoholic steatohepatitis, weighted gene co-expression network analysis, hub genes, immune response, transcription factors

## Abstract

**Introduction:**

Non-alcoholic steatohepatitis (NASH), an advanced subtype of non-alcoholic fatty liver disease (NAFLD), has becoming the most important aetiology for end-stage liver disease, such as cirrhosis and hepatocellular carcinoma. This study were designed to explore novel genes associated with NASH.

**Methods:**

Here, five independent Gene Expression Omnibus (GEO) datasets were combined into a single cohort and analyzed using network biology approaches.

**Results:**

11 modules identified by weighted gene co-expression network analysis (WGCNA) showed significant association with the status of NASH. Further characterization of four gene modules of interest demonstrated that molecular pathology of NASH involves the upregulation of hub genes related to immune response, cholesterol and lipid metabolic process, extracellular matrix organization, and the downregulation of hub genes related to cellular amino acid catabolic, respectively. After DEGs enrichment analysis and module preservation analysis, the Turquoise module associated with immune response displayed a remarkably correlation with NASH status. Hub genes with high degree of connectivity in the module, including CD53, LCP1, LAPTM5, NCKAP1L, C3AR1, PLEK, FCER1G, HLA-DRA and SRGN were further verified in clinical samples and mouse model of NASH. Moreover, single-cell RNA-seq analysis showed that those key genes were expressed by distinct immune cells such as microphages, natural killer, dendritic, T and B cells. Finally, the potential transcription factors of Turquoise module were characterized, including NFKB1, STAT3, RFX5, ILF3, ELF1, SPI1, ETS1 and CEBPA, the expression of which increased with NASH progression.

**Discussion:**

In conclusion, our integrative analysis will contribute to the understanding of NASH and may enable the development of potential biomarkers for NASH therapy.

## Introduction

Non-alcoholic fatty liver disease (NAFLD) is likely to become the most common chronic liver disease, affecting about 25% in the adult population (1). It is characterized by excessive accumulation of hepatic triacylglycerol (TG) and encompasses a spectrum of liver pathologies ranging from isolated steatosis (non-alcoholic fatty liver, NAFL) to non-alcoholic steatohepatitis (NASH), a more severe form of fatty liver disease featured by lobular inflammatory infiltrates, hepatocyte ballooning and fibrosis (2). Up to 30% of the patients with NAFLD will process to NASH (3), which may eventually progress to cirrhosis, hepatocellular carcinoma (HCC) and liver failure (4). Moreover, NASH is considered the hepatic manifestation of metabolic syndrome, commonly alongside serious extrahepatic diseases, such as dyslipidemia, hypertension, obesity and type 2 diabetes mellitus (T2DM) (5, 6), and multiple pathogenic pathways are involved in NASH progression.

Previous studies have contributed greatly to our understanding of genetic and environmental risk factors in the pathogenesis of NAFLD. Genome-wide association studies (GWAS) have revealed genetic variants in several loci (*PNPLA3*, *TM6SF2*, *GCKR*, *MTARC1* and *HSD17B13*) that promote NAFLD risks in humans (7–11), which highlights the dysregulation of gene expression and/or function as an important players in the development and progression of NASH. Integrating multi-omics approaches including genomics, transcriptomics, proteomics and metabolomics have provided additional insights (12–15), which may not be elucidated by genomics analysis alone. In addition, previous bioinformatics analyses in cross-sectional studies have facilitated the exploration of potential biomarkers related to NAFLD/NASH (16–19). However, for complex disease trait, the comprehensive molecular characterization of NASH are still not entirely deciphered. As a consequence, no effective pharmacological therapies targeting NASH are presently available. Hence, further exploration into the molecular pathogenesis of NASH and diagnostic biomarkers are essential to build novel approaches for management of NASH.

Network biology approaches have proven effective for uncovering new perturbed pathways underlying molecular pathology (18, 20, 21). Contrary to traditional differential expression analysis methods based on gene expression profiling, network-based approaches investigate the correlation among changing genes from a systematic perspective. Weighted gene co-expression network analysis (WGCNA) has become a frequently used method for multigene analysis, which establishes gene sets (modules) from observed gene expression data using unsupervised hierarchical clustering. WGCNA is widely used for exploring the relationship between diverse gene sets and clinical features (22, 23), providing insights into functions of co-expression gene modules and detecting hub genes related to the clinical characteristics of various diseases (24, 25).

In the present work, we aimed to identify deregulated modules, hub genes and transcription factors (TFs) associated with NASH by integrating transcriptomic data with biological network analysis between normal liver tissues and NASH tissues. We obtained five liver transcriptome datasets from the Gene Expression Omnibus (GEO) database (26). We first generated MergeCohort by merging five pre-processed datasets. Based on the combining expression matrix, differentially expressed gene (DEG) analysis was performed to identify genes associated with NASH. After that, through integrative analyses of co-expression gene network, functional annotation, TF-target regulatory network and validation analysis, we detected several promising candidate biomarkers for NASH. Our integrative study provides a comprehensive view on the molecular processes of NASH and may discover potential therapeutic target for NASH treatment.

## Methods

### Data collection

**Figure 1:**
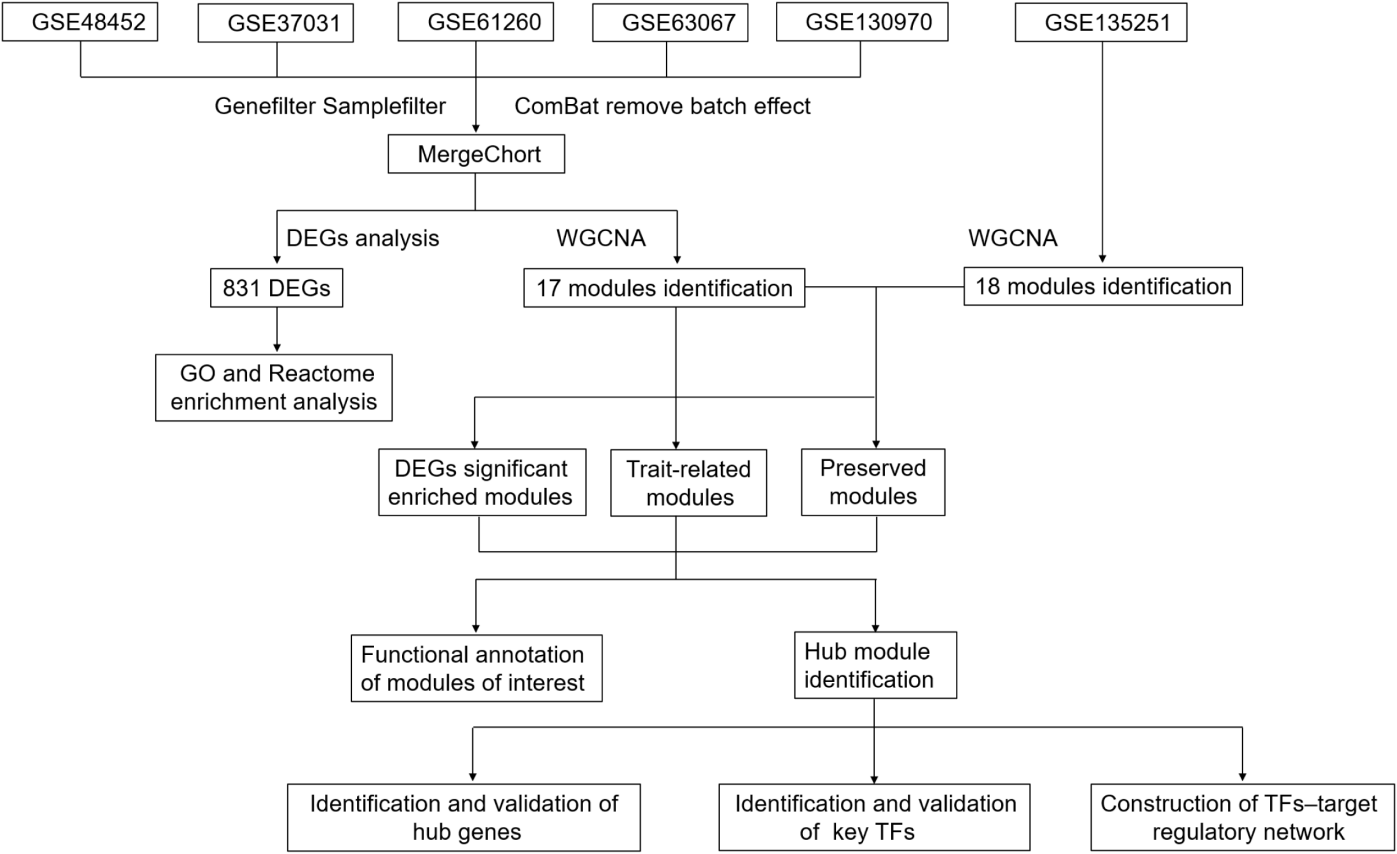
Flowchart.

We obtained the expressing profiles of mRNA of NASH and normal control from the Gene Expression Omnibus (GEO) database (http://www.ncbi.nlm.nih.gov/) (26). We searched the microarray and next-generation sequencing (NGS) studies with the keywords: “Fatty liver”, “Non-alcoholic”, “Gene expression”, “Homo sapiens”, “Microarray” and “RNA sequencing”. Datasets were selected based on the following criterial (1): Containing at least 10 total samples (2); Samples must Contain at least five patients in both NASH group and healthy control group (3); Raw data or gene expression profiles were available in GEO (4). Pathways related to lipid metabolism, inflammation and fibrosis were significantly (normalized enrichment score (NES) more than 1.0 and a false discovery rate (FDR) below 0.25) enriched between the two groups in the gene set enrichment analysis (GSEA) (**Supplementary Tables S2, S3**), which was carried out with the Java GSEA (version 3.0) (27) platform with the ‘Signal2Noise’ metric to create a ranked list and a ‘gene set’ permutation type. The flowchart was shown in **Figure 1**.

### Data processing

For each dataset, we download raw expression data and pre-processed using standard approaches. Specially, gene chip datasets were normalized by the robust multi-average (RMA) method with oligo/Bioconductor (28). For RNA-seq datasets, reads count information were generated by StringTie using a Python script (prepDE.py) and raw counts were normalized across samples following TMM method in edgeR package. After filtering low abundance expression genes and outlier samples, we applied the ComBat (version 3.20.0) method in the sva R package to remove the batch effects (29) from five datasets (GSE48452, GSE37031, GSE61260, GSE63067 and GSE130970) and combined these five datasets into a single cohort (MergeCohort), which contains 67 normal and 97 NASH tissue samples. Subsequently, the expression matrix of MergeCohort was used for differentially expressed genes (DEGs) identification between NASH and healthy control samples. It is worth noticing that we applied Wilcoxon’s rank-sum test to assess the differential expression, the corrected threshold was *p* less than 0.05, and the absolute difference of means more than 0.3. Gene ontology (GO) and Reactome enrichment analyses were performed for DEGs using hypergeometric test, which is conducted by the python package gseapy (version 0.9.16; https://github.com/zqfang/gseapy), all gene sets of GO term and Reactome pathway were obtained from database source of Enrichr (30). Only GO terms or Reactome pathways were considered as significantly enriched by using the criterion with a corresponding *p* value less than 0.05.

### Weight gene co-expression network construction, module detection and preservation analysis of theco-expression modules

5,000 transcripts with maximal variability across all patients (*n* = 164) based on the median absolute deviation in the MergeCohort were kept for WGCNA and tested by the WGCNA R package (22). In our work, the power threshold of 5 was selected to calculate biweight midcorrelations and weighted adjacency matrix, the soft thresholding parameter was defined using the scale-free topology fit model. We identified the gene modules based on the ‘hybrid’ method and parameters deepSplit = 4, mergeCutHeight = 0.15 and minModuleSize = 50. Modules are identified as branches in the dendrogram with Dynamic Tree Cut algorithm (22). Subsequently, we assessed the relevance of a module eigengene (ME) to the disease status using the Pearson correlation. An intramodular connectivity (K_in_) was defined to measure for each gene on the base of its correlation with the remaining genes in a given module. Genes with highest K_in_ are identified as hub genes. Cytoscape version 3.8.2 was used for visualization. In order to understand the extent of module preservation in MergeCohort, a publicly available expression profiling of high throughput RNA sequencing dataset GSE135251 including 10 controls, 51 NAFL and 155 NASH was used, processed as described above. Module preservation analysis was carried out by using Module preservation function in WGCNA package introduced by Langfelder et al. (31) and described in detail in Oldham et al. (32). Moreover, to investigate the module similarity among different cohorts, we applied hypergeometric test to evaluate whether the genes from each MergeCohort module significantly overlapped with the genes from each of GSE135251 module. The overlap was regarded as significant when *p* value below 0.05.

### Functional annotation of the modules

In order to determine the functional significance of the identified modules, we firstly performed GO and KEGG pathway enrichment analysis for the gene lists of each module of co-expression network on the basis of Enrichr (30) as described above. Moreover, we carried out disease enrichment analysis for the gene lists of each module by using DisGeNet (33). The statistical significance threshold level for all disease terms was *p* value less than 0.05 (Benjamini-Hochberg corrected for multiple comparisons) and we presented top 20 for each disease-associated module. Additionally, to obtain regulatory information of transcription factors (TFs) and target genes, Transcriptional Regulatory Relationships Unraveled by Sentence based Text mining (TRRUST) v2 database (https://www.grnpedia.org/trrust/) (34) were supplied for Enrichr (30), conducted by the python package gseapy (version 0.9.16; https://github.com/zqfang/gseapy). In addition, ChIP-X Enrichment Analysis 3 (ChEA3) database (https://maayanlab.cloud/chea3/) (35) was adopted to further validate the significantly enriched transcription factors over module genes. After obtaining TF–target regulatory relationships, a TF-target network, which contained TFs regulating Turquoise modules’ genes, was reconstructed.

### Single cell RNA-sequencing analyses

We investigated the expression patterns of top 25 hub genes in Turquoise module using scRNA-seq analyses of human liver tissues from public scRNA-seq data (GSE136103) (36). In our study, only four samples including two healthy liver tissue samples (GSM4041156 and GSM4041159) and two NAFLD liver tissue samples (GSM4041162 and GSM4041163) were analyzed with Seurat package (version 3.1.5) (37). First, 2000 highly variable genes (*n* = 2,000) were identified using the R package *SCTransfom* (version 0.2.1). Subsequently, principal component analysis was performed, and the appropriate principal components (PCs) for dimensionality reduction were decided using the *JackStraw* function. Clusters were identified with the Seurat function *FindClusters* with the resolution set at 0.4. This method resulted in 18 clusters, which were visualized by Uniform Manifold Approximation and Projection (UMAP) analysis. Clusters were then annotated by using the expression of known genes. We annotated cell types based on cell markers and the R package SingleR (36, 38).

## Results

### Information of included GEO datasets

According to the previously established inclusion criteria, GSE48452, GSE37031, GSE61260, GSE63067 and GSE130970 were included in this study. There are 104 NASH patients and 70 controls in these five datasets. After outlier removal, 97 NASH patients and 67 controls were retained in the following analysis. The detail information of the five datasets was shown in **Supplementary Table S1**. In order to eliminate the bath effect from different platforms and batches, we used the combat function to eliminate the batch effect from five datasets. A total of 12579 genes were detected by merging different platforms. Before removing the batch effect, samples were clusters in batch according to the top two principal components (PCs) of the expression values before normalization (**Figure S1A**). In contrast, when the samples from five platforms were merged, the overall expression in the samples was uniformly distributed based on principal component analysis, suggesting that the batch effect caused by different platforms that had effect on the estimation of molecular biological differences was successfully corrected (**Figure S1B**). In addition, we used dataset GSE135251 as the validation dataset in this study.

### Identification of DEGs in the NASH patients

**Figure 2:**
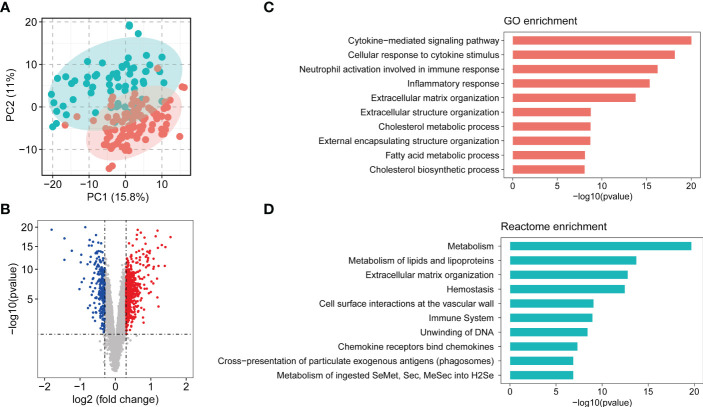
Overview of combining gene expression profiles in healthy controls and nonalcoholic steatohepatitis (NASH) patients. **(A)** Principle component plot of samples based on top 500 most variable gene expression from combining gene expression profiles (MergeCohort). NASH patients are marked in red; healthy controls are marked in green. **(B)** Volcano plot of differentially expressed genes (DEGs) between NASH patients and healthy controls. DEGs are listed in **Supplemental Table S4**. 600 genes upregulated and 200 genes downregulated are shown in red and blue, respectively. **(C)** Top 10 enriched biological functions of DEGs determined by Gene Ontology (GO) enrichment analysis. **(D)** Top 10 enriched Reactome pathways of DEGs determined by Reactome pathway enrichment analysis.

Principle component analysis plot of the gene expression matrix of five combined dataset (MergeCohort) distinguished between NASH and control group is shown in **Figure 2A**. Total of 831 DEGs (Benjamin-Hochberg adjusted *p* value < 0.05, absolute difference of mean > 0.3) among control and NASH in MergeCohort were identified, consisting of 600 upregulated and 231 downregulated DEGs (**Figure 2B**; **Supplementary Table S4**).

### Function and pathway enrichment analysis of DEGs

In the present study, we performed GO and Reactome pathway enrichment analysis to determine the potential functions of 831 DEGs in the pathogenesis of NASH. The biological process analysis (**Figure 2C**; **Supplementary Table S5**) revealed that in the NASH, these genes were associated with multiple immunity-related pathways, such as the cytokine-mediated signaling pathway, cellular response to cytokine stimulus and neutrophil activation involved in immune response. Several ECM-related pathways were also enriched such as extracellular matrix organization and extracellular structure organization. Moreover, metabolic process, such as cholesterol metabolic process, fatty acid metabolic process, cholesterol biosynthetic process and other biological process (**Supplementary Table S5**) were also identified. Reactome pathway analysis was performed to investigate the pathway based on the DEGs (**Supplementary Table S6**). The top 10 pathways are shown in **Figure 2D**. Among them, metabolism, metabolism of lipids and lipoproteins, extracellular matrix organization, immune system, chemokine receptors bind chemokines were significantly enriched. Therefore, the outcomes above suggested that metabolism, ECM-related pathways and immunity-related pathways play an important role in development and procession of NASH.

### WGCNA and identification of module associated with NASH disease status

**Figure 3:**
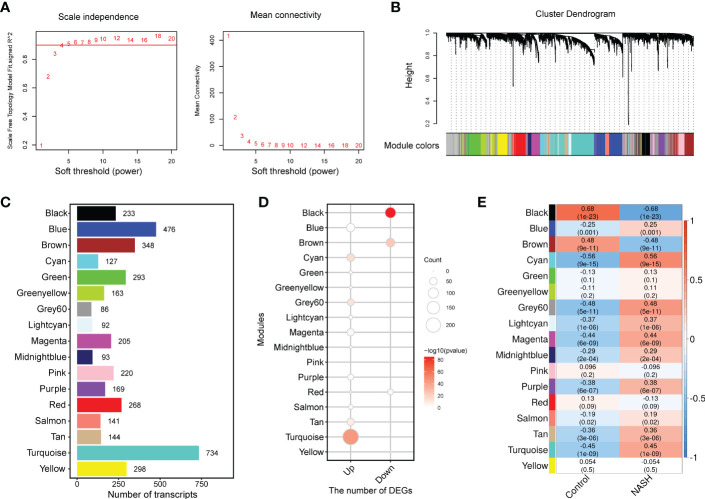
WGCNA network and module identification. **(A)** Soft-thresholding calculation of MergeCohort. The left panel displays the scale-free fit index versus soft-thresholding power. The right panel shows the mean connectivity versus soft-thresholding power. Power 5 was selected, for which the fit index curve flattens out upon reaching a high value (> 0.9). **(B)** The Cluster dendrogram of co-expression network modules from WGCNA depending on a dissimilarity measure (1-TOM). The leaves in the tree represent genes and the colors in the horizontal bar indicate co-expression module determined by the dynamic tree cut algorithm. **(C)** Number of genes in each module. **(D)** Enrichment of upregulated and downregulated DEGs in each module. **(E)** Heatmap showing the association between module eigengenes (rows) and NASH disease status (column). Associated *p* values were computed using the cor.test R function. The color scale in the heat map represents the magnitude of the Pearson correlation coefficients. Number in each cell contained corresponding correlation coefficient and *p* value (in brackets). WGCNA, weighted gene correlation network analysis; TOM, topological overlap matrix.

To capture discrete groups of co-expression genes correlated with NASH status and to integrate the identified expression divergences into a higher system level context, a co-expression network analysis (WGCNA) was conducted based on the top 5000 median absolute deviation (MAD) genes from the MergeCohort. Keep to the scale-free topology criterion, β=5 was considered in this study (**Figure 3A**). According to dynamic tree cut, the hierarchical clustering dendrogram resulted in 17 different gene modules, as displayed in **Figure 3B**. 909 genes failed to fit within a distinct group and were assigned to the Grey module which was neglected in the present study. The size of modules ranged from 86 (Grey60 module) to 734 (Turquoise module) (**Figure 3C**). DEGs enrichment in each module was shown in **Figure 3D**, in which upregulated genes was mostly significantly enriched in Turquoise (*n* = 233, *p* = 1.93 × 10^-44^), and followed by Cyan (*n* = 54, *p* = 1.24 × 10^-15^), Grey60 (*n* = 40, *p* = 2.05 × 10^-13^), Tan (*n* = 48, *p* = 1.59× 10^-9^) and Magenta (*n* = 47, *p* =2.77 × 10^-4^), downregulated genes was significantly enriched in Black (*n* = 107, *p* = 9.25 × 10^-86^) and Brown module (*n* = 68, *p* = 1.07 × 10^-24^). To investigate which co-expression modules are associated with NASH status, we then correlated the expression of eigengenes (genes representing the expression profile of each module) with NASH status. The relationship between all the modules and the NASH status are displayed in a correlation heatmap, in which Y-axis corresponds to groups of genes (modules) and the X-axis represents the NASH status (**Figure 3E**). Of the 17 co-expression modules, 11 WGCNA modules to be correlated with NASH status at a Pearson correlation (*p* < 1.47 × 10^-3^), which is determined based on Bonferroni correction. Among them, nine modules (Cyan, Grey60, Turquoise, Magenta, Purple, Lightcyan, Tan, Midnightblue and Blue) were positively correlated with NASH disease status, two modules (Black and Brown) were negatively associated with NASH disease status (**Figure 3E**).

### Functional characterization of co-expression modules of interest

**Figure 4:**
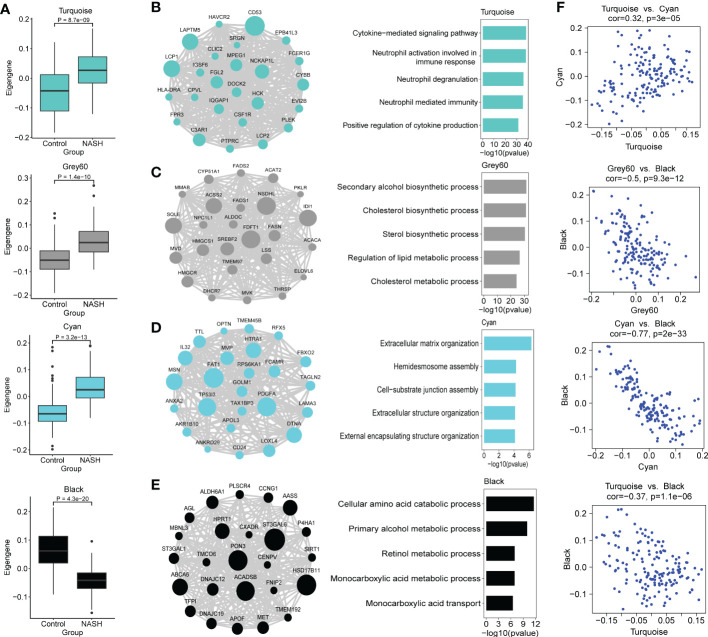
Functional characterization of co-expression modules of interest identified by WGCNA. **(A)** Box and Whisker plots representing the expression of module eigengenes Turquoise, Grey60, Cyan, Black between NASH (*n* = 97) and healthy control (*n* = 67) samples. Data are presented as median with first and third quartiles as the box edges. Differences between group were estimated by Student’s *t* test. **(B–E)** The network of hub genes (module genes within the top 25 genes with the highest intromodular connectivity values (kWithin)) (left panel) and top GO terms (right panel) of the modules Turquoise **(B)**, Grey60 **(C)**, Cyan **(D)** and Black **(E)** are shown. In the network diagrams, node sizes correspond to kWithin in the module. For the bars plot, the bars in the GO enrichment results represent the -log_10_(pvalue). **(F)** Scatterplots of module eigengenes show positive correlation between Turquoise and Cyan, and negative correlation between Grey60, Cyan, Turquoise and Black, respectively.

Because we were more concerned about the modules whose expression was different between NASH and control group, we compared the eigengenes from NASH samples to the expression of control in every module, and these results were used to further assess whether the modules were associated with NASH status. Modules Cyan, Grey60 and Turquoise exhibited an upregulation of the eigengenes in NASH, whereas module black showed lower expression in NASH (**Figure 4A**). In order to investigate whether the co-expression modules cover the information associated with validated networks, the existing data on protein-protein interactions from the STRING database was used to test the biological characteristics of the detected modules in this study. All the modules showed significant enrichment in interactions (*p* < 0.01), therefore indicating that the modules detected in the present work are biologically relevant (**Supplementary Table S7**). In addition, the NASH status positively correlated modules showed much higher average node degree (AND), particularly module Turquoise (AND = 22.4).

We then conducted GO and KEGG pathway enrichment of the NASH-associated modules to further investigate the gene functions by Enrichr. Top biological process and KEGG pathway in each module are shown in **Table 1**. Turquoise module was upregulated in NASH patients, contained hub genes related to immune response (*CD53*, *LAPTM5*, *LCP1*, *NCKAP1L*, *C3AR1* and *FGL2*) (**Figure 4B**), and enriched for GO categories to cytokine-mediated signaling pathway, neutrophil activation involved in immune response and neutrophil degranulation (**Figure 4B**). Grey60 module with hub genes such as *FDFT1*, *NSDHL*, *IDI1*, *SQLE*, *ACSS2*, *SREBF2*, *HMGCR*, *FASN*, *LSS*, *ACAT2*, *FADS1*, *FADS2* and *ELOVL6* was upregulated in NASH (**Figure 4C**), which were mainly participating in cholesterol and lipid metabolic process (**Figure 4C**). The majority of the GO terms enriched in module Cyan were primarily related to extracellular matrix organization and extracellular structure organization (**Figure 4D**), including hub genes related to fibrosis (*PDGFA*, *LOXL4*, *MSN*, *LAMA3* and *AKR1B10*) (**Figure 4D**). However, the majority of the GO terms enrich in Black module were related to cellular amino acid catabolic and primary alcohol metabolic process (*ACADSB*, *AASS* and *ALDH6A1*) (**Figure 4E**). The complete annotation for each module can be found in **Supplementary Tables S8, S9**.

**Table 1:** Top GO and pathway enrichment in each module.

Module	Category	Term	*P*-value	FDR
Black	GOTERM_BP	Cellular amino acid catabolic process	2.37 × 10^-12^	3.95 × 10^-09^
Blue	GOTERM_BP	Extracellular matrix organization	6.18 × 10^-37^	1.57 × 10^-33^
Brown	GOTERM_BP	Cellular amino acid catabolic process	5.27 × 10^-09^	1.06 × 10^-05^
Cyan	GOTERM_BP	Extracellular matrix organization	4.82 × 10^-07^	5.88 × 10^-04^
Grey60	GOTERM_BP	Secondary alcohol biosynthetic process	2.39 × 10^-32^	1.54 × 10^-29^
Lightcyan	GOTERM_BP	T cell activation	4.17 × 10^-13^	3.44 × 10^-10^
Magenta	GOTERM_BP	DNA metabolic process	2.69 × 10^-45^	3.48 × 10^-42^
Midnightblue	GOTERM_BP	IRE1-mediated unfolded protein response	7.75 × 10^-16^	6.39 × 10^-13^
Purple	GOTERM_BP	Regulation of glycogen metabolic process	2.31 × 10^-06^	3.06 × 10^-03^
Tan	GOTERM_BP	Neutrophil degranulation	8.86 × 10^-16^	7.05 × 10^-13^
Turquoise	GOTERM_BP	Cytokine-mediated signaling pathway	3.47 × 10^-39^	8.55 × 10^-36^
Black	KEGG_PATHWAY	Metabolism of xenobiotics by cytochrome P450	2.94 × 10^-05^	3.85 × 10^-03^
Blue	KEGG_PATHWAY	ECM-receptor interaction	3.54 × 10^-19^	8.42 × 10^-17^
Brown	KEGG_PATHWAY	Glycine, serine and threonine metabolism	2.24 × 10^-08^	5.78 × 10^-06^
Cyan	KEGG_PATHWAY	Mitophagy	9.22 × 10^-04^	0.11
Grey60	KEGG_PATHWAY	Steroid biosynthesis	1.01 × 10^-14^	8.99 × 10^-13^
Lightcyan	KEGG_PATHWAY	Primary immunodeficiency	1.14 × 10^-17^	1.39 × 10^-15^
Magenta	KEGG_PATHWAY	DNA replication	5.62 × 10^-27^	7.20 × 10^-25^
Midnightblue	KEGG_PATHWAY	Protein processing in endoplasmic reticulum	3.05 × 10^-21^	2.75 × 10^-19^
Purple	KEGG_PATHWAY	Axon guidance	1.62 × 10^-04^	3.11 × 10^-02^
Tan	KEGG_PATHWAY	Cytokine-cytokine receptor interaction	4.47 × 10^-12^	8.81 × 10^-10^
Turquoise	KEGG_PATHWAY	Osteoclast differentiation	2.48 × 10^-18^	6.45 × 10^-16^

We next explored the relationship of eigengenes among the annotated modules. Upregulated immune Turquoise module was positively correlated with Cyan module related to fibrosis (r = 0.32, *p* = 3.0 × 10^-5^) (**Figure 4F**), suggesting that Turquoise module related to immune response that drives fibrosis in NASH, which confirmed the results of previous studies (20). Interestingly, Cyan, Grey60 and Turquoise modules was negatively correlated with Black module that is enriched in amino acid metabolic processes (**Figure 4F**). The high negatively correlation (r = -0.77, *p* = 2.0 × 10^-33^) between the upregulated fibrosis module Cyan and downregulated Black module that is enriched in metabolic processes (**Figure 4F**), which indicated that perturbations in amino acid metabolism are likely involved in NASH pathogenesis (39, 40).

### Module preservation analysis indicates the presence of NASH-associated co-expression module function in immune response

**Figure 5:**
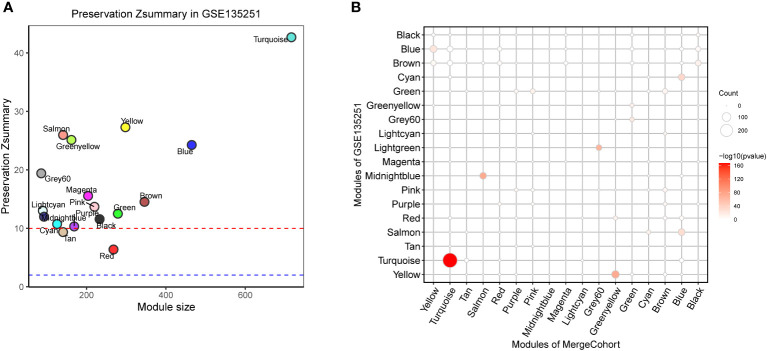
Module preservation of MergeCohort in GSE135251 dataset. **(A)** Preservation Zsummary statistics of MergeCohort in GSE135251 dataset. Each point represents a module. Point color reflects the module color as used in **Figures 3B–E** of MergeCohort. Points are also labeled by the name of the module. The dashed blue and red lines indicate the rough thresholds for week (Z = 2) and strong (Z = 10) evidence of module preservation. **(B)** Overlaps of MergeCohort and GSE135251 modules. Each axis is labelled by the corresponding module name. The size of each dot represents the number of overlapping genes in the intersection of corresponding MergeCohort and GSE135251 modules while the color implies -log_10_ of the hypergeometric enrichment *p* value.

**Figure 6:**
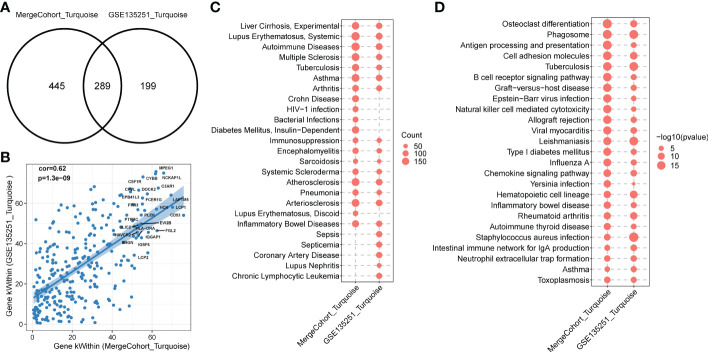
Functional enrichment of MergeCohort_Turquoise and GSE135251_Turquoise module. **(A)** Venn diagram displays number of genes overlapped between MergeCohort_Turquoise and GSE135251_Turquoise module. **(B)** Spearman’s correlation between the kWithin of common genes (*n* = 289) overlapped between each module. Top 25 hub genes with the highest kWithin from MergeCohort_Turquoise module are shown. **(C)** Dot-plot heatmap shows top 20 significantly enriched disease by genes in each module. The size of each dot represent the gene counts enriched in each disease term. **(D)** Dot-plot heatmap shows top 20 significantly enriched KEGG pathways by genes in each module. The size of each dot represents the -log_10_ of *p* value for each KEGG pathway term.

To find out whether the identified modules were common in another dataset, we examined the module preservation statistics between the MergeCohort and one recently published large NASH datatset GSE135251 (13). In particular, we assumed co-expression modules of MergeCohort as reference dataset and the co-expression modules of GSE135251 as test dataset. We utilized the principle described in (22). The score of Zsummary more than 10 represents strongly preserved module, less than 2 denotes non-preserved module while the value between 2 and 10 implies moderately preserved module. We plotted the scatterplot of Zsummary scores against the sizes of MergeCohort modules (**Figure 5A**). All modules have a Zsummary statics greater than 2, suggesting that all modules were preserved in GSE135251. The lowest preservation is the Red module (Zsummary = 6.37). Particularly, MergeCohort module Turquoise (MergeCohort_Turquoise) exhibited Zsummary preservation score (Zsummary = 42.68) higher than 40. To provide a more intuitive picture of the preservation of each co-expression module identified, we evaluated module overlaps of MergeCohort and GSE135251 (**Figure 5B**), we found that MergeCohort_Turquoise show the most significantly overlapping with GSE135251 module Turquoise (GSE135251_Turquoise). Moreover, we discovered a highly positively correlation between the intromodular connectivity of 289 genes overlapped in MergeCohort_Turquoise and GSE135251_Turquoise (Spearman’s correlation = 0.62, *p* = 1.3 × 10^-9^) (**Figures 6A, B**), which indicated those two modules have similar co-expression pattern.

To comprehensively evaluate the biological functions related to MergeCohort_Turquoise and GSE135251_Turquoise, we next calculated the statistical significance of enrichment of genes with the association in disease-related gene sets from the DisGeNET database (33) and KEGG pathway gene sets. We observed that genes in MergeCohort_Turquoise and GSE135251_Turquoise were significantly enriched by liver disease-related gene sets (liver cirrhosis) and multiple immune disease-related gene sets (autoimmune disease, immunosuppression and inflammatory bowel disease) (**Figure 6C**; **Supplementary Tables S10, S11**). Interestingly, these two modules were also significantly enriched in atherosclerosis and arteriosclerosis. Notably, we observed that genes in MergeCohort_Turquoise, which shows the highest module similarity with GSE135251_Turquoise (289 out of 734; hypergeometric test *p* value = 5.33 × 10^-168^) (**Figure 6A**) are both significant enriched in phagosome, osteoclast differentiation, cell adhesion molecules, antigen processing and presentation, B cell receptor signaling pathway (**Figure 6D**). In addition, the MergeCohort_Turquoise was upregulated in NASH and is also the third most significant module, and showed the greater number of statistically differential expressed genes, with 233 of the 734 genes being upregulated (fold change > 1.2; *p* < 0.05) and none significantly downregulated (**Figure 3D**). Considering all these results, we will choose the co-expression Turquoise module from MergeCohort for further analysis.

### Validation of hub genes in Turquoise module

**Figure 7:**
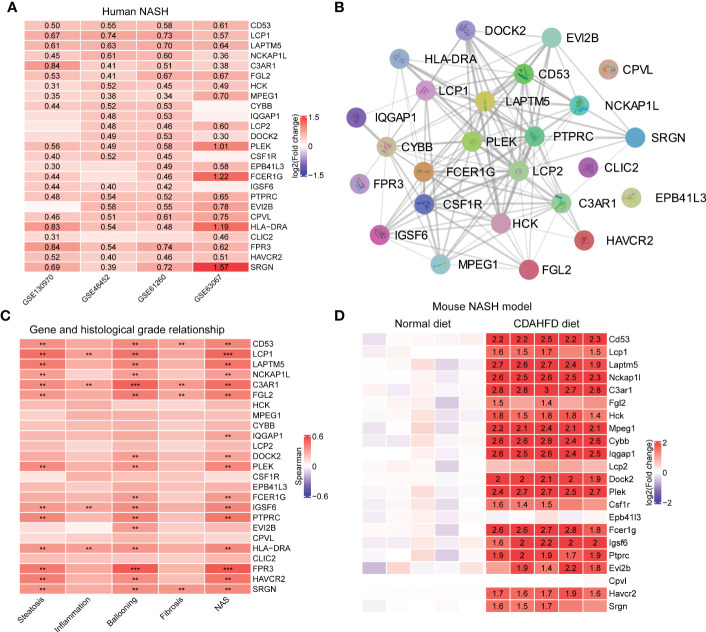
Validation of hub genes in MergeCohort_Turquoise module. **(A)** Heatmap shows the expression patterns of top 25 hub genes in human liver tissues according to four datasets (GSE130970, GSE48452, GSE61260 and GSE63067). The numbers in heatmap represent log_2_ value of fold change between NASH patients and healthy controls. **(B)** The protein-protein interactions among top 25 hub genes were retrieved by the STRING database. **(C)** Heatmap shows the Person correlation coefficients of top 25 hub genes and clinical parameters of NAFLD according to GSE130970 dataset. *p* values are overlaid on the heatmap (^**^ *p* < 0.01 and ^***^ *p* < 0.001). **(D)** Heatmap shows the expression patterns of top 25 hub genes in mouse liver tissue according to GSE120977 dataset. The numbers in heatmap represent log_2_ value of fold change between the CDAHFD and chow diet control group. CDAHFD, choline deficient L-amino acid defined high fat diet.

Hub genes were upregulated in the liver from NASH patients. Focusing on the MergeCohort_Turquoise module, we firstly explored the top 25 hub genes including *CD53*, *LCP1*, *LAPTM5*, *NCKAP1L*, *C3AR1*, *PLEK*, *FCER1G*, *HLA-DRA* and *SRGN* that had a high intramodular connectivity (K.in). The expression level of those core genes were all upregulated in four cohorts (GSE130970, GSE48452, GSE61260 and GSE63067) involved in this study **Figure 7A**, suggesting that these hub genes may play fundamental role in NASH development. The PPI network of these 25 hub genes was showed in **Figure 7B**.

Hub genes were positively correlated with clinical characteristics. We further investigated the relationship between the changes in expression of these 25 hub genes and the histological phenotype in GSE130970 (**Figure 7C**). Our results demonstrated that each of the 25 key genes were positively correlated with the NAFLD activity score, and *FPR3* has the highest correlation (r = 0.53, *p* = 1.49 × 10^-4^). *LCP1* gene was the most associated gene with steatosis grade (r = 0.46, *p* = 1.16 × 10^-3^) and the lobular inflammation grade (r = 0.32, *p* = 3.06 × 10^-2^). Moreover, *FPR3* associated most with the cytological ballooning grade (r = 0.53, *p* = 1.82 × 10^-4^). *SRGN* was the most relevant gene with the fibrosis stage (r = 0.35, *p* = 1.84 × 10^-2^). Additionally, *C3AR1* showed significant correlation with all the clinical parameters, especially higher correlation with the cytological ballooning grade (r = 0.51, *p* = 2.94 × 10^-4^).

Hub genes were upregulated in the liver from the choline deficient L-amino acid defined high fat diet (CDAHFD) model of NASH in mouse. Furthermore, to explore the significance of the hub genes in mouse, we mined public available microarray data (GSE120977) (41) to validate the mRNA levels of the abovementioned genes, except *Hla-dra*, *Clic2* and *Fpr3* gene which was lacking in the dataset. Intriguingly, several of the hub genes displayed either a significant or a trending higher expression in mouse individuals fed with CDAHFD diets at 12 weeks compared with the controls. For instance, 14 genes, namely *Cd53*, *Laptm5*, *Nckap1l*, *C3ar1*, *Hck*, *Mpeg1*, *Cybb*, *Iqgap1*, *Dock2*, *Plek*, *Fcer1g*, *Igsf6*, *Ptprc* and *Havcr2*, which were strongly upregulated in mouse fed with CDAHFD chow (**Figure 7D**), supporting the notion that these hub genes were also activated during progression of mouse NASH model.

### Identification of cell clusters contributions to the NASH-associated Turquoise module integrating single-cell RNA-seq analysis

**Figure 8:**
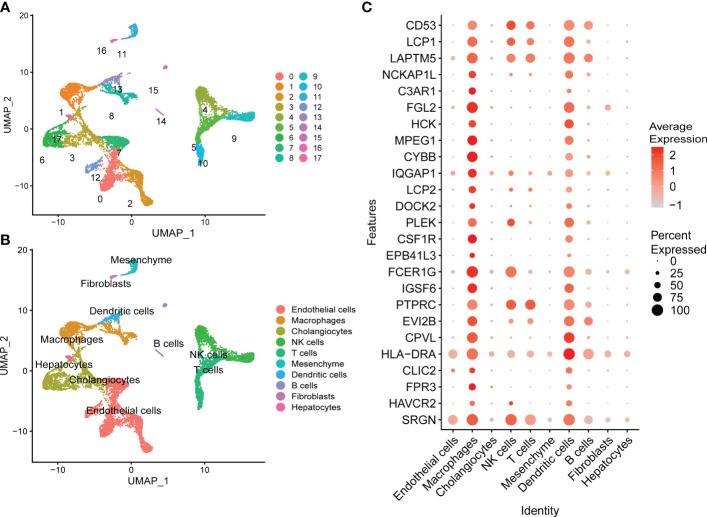
Assessment of the expression patterns of hub genes in MergeCohort_Turquoise module in different types of cells using publicly available healthy and cirrhotic scRNA-seq from dataset GSE136103. **(A)** UMAP visualization of different cell clusters from healthy (*n* = 2) and cirrhotic (*n* = 2) human livers. **(B)** UMAP visualization of cell types from healthy (*n* = 2) and cirrhotic (*n* = 2) human livers. Cells were annotated as endothelial cells, macrophages, cholangiocytes, NK cells, T cells, mesenchyme, dendritic cells, B cells, fibroblasts, and hepatocytes based on the expression of lineage markers. **(C)** Dot plot shows the expression patterns of top 25 hub genes in different types of liver cells. Size of the dot indicates proportion of the cell population that expresses each gene. Color represents level of expression. UMAP, uniform manifold approximation and projection.

To investigate how potential hub genes identified in MergeCohort_Turquoise module change within specific cell populations during NASH progression, we carried out an integrated scRNA-seq analysis using publicly available scRNA-seq data from healthy and cirrhotic liver samples. Clustering revealed 17 populations of cells comprising 10 distinct cell types (**Figures 8A, B**; **Supplementary Figure S2**). We identified Endothelial cells, macrophages, cholangiocytes, NK cells, T cells, mesenchyme, dendritic cells, B cells, fibroblasts, and hepatocytes within the scRNA-seq data based on the expression of lineage specific markers as annotated with integration of discoveries from human liver cell atlas and the annotation analysis with SingleR. The expression patterns of the top 25 genes in the MergeCohort_Turquoise module were analyzed by scRNA-seq analyses of liver tissues. Those key genes in MergeCohort_Turquoise module including *CD53*, *LCP1*, *LAPTM5*, *PTPRC* and *SRGN* expressed by distinct immune cells such as microphages, NK cells, T cells, dendritic cells and B cells, and most of them, namely *FGL2*, *HCK*, *MPEG1*, *CYBB*, *CSF1R*, *IGSF6*, *CPVL* and *HLA-DRA* were mainly expressed by macrophages, dendritic cells (**Figure 8C**; **Supplementary Figure S3**), which indicated that the macrophages and dendritic cells play an important role in the pathogenesis of NASH.

### Identification of TFs that regulate the Turquoise modules

**Figure 9:**
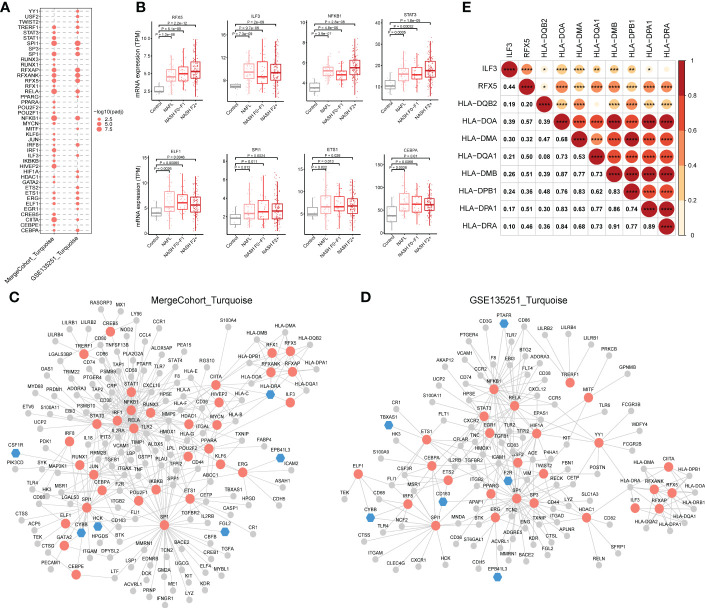
Regulatory relationship between enriched transcription factors and their target genes in NASH-associated module. **(A)** Dot-plot heatmap shows enriched transcription factors in MergeCohort_Turquoise and GSE135251_Turquoise module. The size of each dot represents the -log_10_ of adjusted *p* value for each transcription factor. **(B)** Boxplots shows mRNA hepatic expression of the enriched transcription factors including *RFX5*, *ILF3*, *NFKB1*, *STAT3*, *ELF1*, *SPI1*, *ETS1* and *CEBPA* according to GSE135251 dataset. The *p* value was calculated by Student’s t test. **(C, D)** The regulatory networks between enriched transcription factors and associated target genes in MergeCohort_Turquoise **(C)** and GSE135251_Turquoise module **(D)**, respectively. Red color represents transcription factors, blue color represents target hub genes, grey color represents other target genes. **(E)** Pearson correlations for mRNA hepatic expression of transcription factors (*RFX5* and *ILF3*) and associated target genes (*HLA-DQB2*, *HLA-DOA*, *HLA-DMA*, *HLA-DQA1*, *HLA-DMB*, *HLA-DPB1*, *HLA-DPA1* and *HLA-DRA*) in GSE135251 dataset. ^*^ *p* < 0.05, ^**^ *p* < 0.01, ^***^ *p* < 0.001 and ^****^ *p* < 0.0001.

The results of the analysis above showed that hub genes in MergeCohort_Turquoise module were enriched in immunity. Because co-expressed genes tend to be co-regulated by the common transcription factors (TFs), we further conducted TFs enrichment analysis (hypergeometric test) using the genes from the MergeCohort_Turquoise and GSE135251_Turquoise modules to obtain key regulatory genes, based on TRRUST database (34). Our results indicated that *NFKB1*, *SPI1*, *RELA*, *CIITA*, *HIVEP2*, *SP1*, *RFXANK*, *RFXAP*, *RFX5*, *IRF1* are the top 10 most significantly enriched TFs in MergeCohort_Turquoise module (**Figure 9A**). Moreover, we adopted ChEA3 database (35) to validate the significantly enriched transcription factors over MergeCohort_Turquoise module genes. As a result, ChEA3 analysis identified 27 of the 33 significant TFs for MergeCohort_Turquoise module genes with TRRUST database, the other six TFs were part of their targets (**Table S12**). We also found that *NFKB1*, *SPI1*, *RELA*, *CIITA*, *SP1*, *RFXANK*, *RFXAP*, *RFX5*, *TRERF1*, *ELF1*, *STAT3*, *ERG*, *ETS1*, *ILF3*, *CEBPA*, *HDAC1* and *IRF8* are significantly enriched TFs in both MergeCohort_Turquoise and GSE135251_Turquoise module (**Figure 9A**). Furthermore, we observed significantly increased of hepatic expression of *RFX5*, *ILF3*, *NFKB1*, *STAT3*, *ELF1*, *SPI1*, *ETS1* and *CEBPA* in NAFL and NASH compared to the control group (*p* < 0.05) (**Figure 9B**).

Next, the regulatory networks were constructed for the enriched TFs and associated target genes in each of the modules (**Figures 9C, D**). We observed that *RFX5* and *ILF3*, an important transcriptional factor mainly expressed in the liver, upregulated from mild to advanced NASH, regulates the expression of genes involved in antigen processing and presentation of exogenous peptide antigen *via* MHC class II, including *HLA-DQB2*, *HLA-DOA*, *HLA-DMA*, *HLA-DQA1*, *HLA-DMB*, *HLA-DPB1*, *HLA-DPA1* and *HLA-DRA*. Notably, the gene expression of *RFX5* and *ILF3* positively correlated with MHCII gene expression (**Figure 9E**). We found 41 genes are regulated by the *NFKB1* transcription factor. As known, *NFKB1* regulates the expression of genes associated with cytokine-mediated signaling pathway (e.g., *TNF*, *CXCL10*, *MMP9* and *TGFB1*) and immune response (e.g., *CD74*, *CD58*, *CD80* and *CD86*) (**Figure 9C**). Moreover, *STAT3* regulates the expression of gene in Wound healing involved in inflammatory response, including *HMOX1*, *TIMP1*, *TGFB1* and *F2R*. Interestingly, *SPI1* regulated gene involved in immune effector process (e.g., *CTSG*, *CD68*, *IFIT3* and *IL18*) including hub genes (*CYBB* and *HCK)* in MergeCohort_Turquoise module. *SP1* regulated gene involved in cell activation (e.g., *TIMP1*, *LTF*, *FGL2* and *LYZ*).

For further analysis the expression of the hub genes and key TFs *in vitro* models of NASH, we retrieved public available RNA-seq data (the RNA-seq data of L02 hepatocytes (PRJNA726826) and murine primary hepatocytes (PRJNA726846) treated with palmitic acid and oleic acid (PAOA) for 0h, 12h and 24h, respectively (42)), we found hub genes (*CD53* and *SRGN*) and key TFs (*NFKB1*, *ELF1* and *EST1*) displayed higher expression in L02 hepatocytes treated with PAOA (**Figure S4A**). Moreover, we observed that hub genes (*Lcp1* and *Fcer1g*) and key TFs (*Ilf3*, *stat3* and *Est1*) showed increased expression in murine primary hepatocytes with PAOA treatment (**Figure S4B**). Together, these TFs and target genes identified in our study provide a promising list for investigators or companies interested in conducting preclinical study into the mechanisms of and treatments for NASH both *in vitro* and *in vivo*.

## Discussion

The global epidemic of NASH is a serious public health problem, the pathogenesis of NASH still remains unclear. Moreover, although liver biopsy currently remains the reference standard for diagnosis of NASH, it is an intrusive operation with risks and many shortcomings. Thus, identifying novel non-invasive biomarkers in NASH is of paramount importance in the prevention and therapy of this disease.

Thanks to the rapid development of high-throughput sequencing technology and gene chip technology, more and more researchers are actively pursuing molecular markers using data mining and analysis of sequencing data or gene chips to the diagnosis and treatment of disease (19, 43, 44). In our study, we analyzed gene expression profiles of NASH patients and normal controls from five independent GEO data sets. The batch of various platforms or batches is removed. DEGs were identified between normal liver tissues and NASH tissues, based on 831 DEGs between Normal-NASH group, we performed GO and Reactome pathway analysis to explore underlying mechanism of NASH. The results showed that enriched pathways were involved in metabolism pathways, inflammatory response and immune response, extracellular matrix organization (**Figures 2C, D**), conforming their association with NASH development and progression.

Subsequently, we constructed a co-expression network and identified 17 different modules by WGCNA, among which 11 modules were significantly associated with the status of NASH. DEG numbers showed a significant enrichment in seven important modules (**Figure 3D**). The results of this study indicated that the identified modules are biologically rational, majority of which are enriched for specific GO terms and KEGG pathways, sharing some commonality with the existing literature. For example, module Black and Brown, are markedly negative correlated with NASH status. Both the Black and Brown were most significantly enriched in cellular amino acid catabolic process. Recent studies showed that deregulation in amino acid metabolism seem to be involved in the appearance of NASH (39, 45). In addition, previous research has demonstrated that lipid metabolism significantly altered during NASH progression (46). Our data found Grey60 module that was significantly upregulated in NASH, enriched in the lipid metabolism pathways, encompassing hub genes related to cholesterol metabolism (*FDFT1*, *NSDHL*, *IDI1*, *SQLE*, *MVD*, *HMGCS1*, *HMGCR* and *LSS*) as well as fatty acid metabolism (*FASN*, *ELOVL6*, *FADS1*, *FADS2*, *ACACA*, *ELOVL6*, *PKLR* and *THRSP*) (**Figure 4C**). Similarly, previous biological network analysis identified cholesterol synthesis genes in human NAFLD (e.g., *FDFT1*, *NSDHL*, *IDI1*, *SQLE*, *MVD*, *HMGCS1* and *HMGCR*) and fatty acid metabolism genes (e.g., *Fasn*, *Thrsp* and *Pklr*) in NAFLD mouse model that were also reported to be deregulated by (47) and (18), respectively. Thus, despite the differences in study design, the three studies coverage on a number of key biological findings.

Inflammation is an important factor driving NASH progression. Our current systematic transcriptomic analysis also highlighted the importance of the Turquoise module in modulating NASH occurrence and development. This study found that the immune-related pathways were mostly enriched in the Turquoise module, which contained the highest number of differentially deregulated genes (**Figure 3D**). Moreover, we demonstrated the highest preservation of the Turquoise module between the MergeCohort and validation dataset GSE135251 (**Figure 5A**). The top hub genes overexpression in NASH samples and linking immune-related pathways belonged to *CD53*, *LCP1*, *LAPTM5*, *NCKAP1L*, *C3AR1*, *FGL2*, *PLEK*, *HLA-DRA*, *FPR3* and *SRGN*, which also showed positive correlation with histological grade (**Figure 7C**). Further validation by mouse NASH model, the expression of *CD53*, *LCP1*, *LAPTM5*, *NCKAP1L*, *C3AR1*, *FGL2*, *PLEK* and *SRGN* were significantly upregulated (**Figure 7D**). The role of *CD53*, *C3AR1*, *NCKAP1L* and *FGL2* genes in regulation of immune responses has recently been proposed in previous studies. CD53 is a member of the tetraspanin membrane protein family that may be involved in transmembrane signal transduction (48). *CD53* has been reported to associate with liver inflammation and insulin sensitivity (49). LAPTM5 is a transmembrane protein which is preferentially expressed in immune cells, and it acts as a positive regulator of proinflammatory signaling pathways in macrophages (50). Previous study revealed that *LAPTM5* could interact with *CDC42*, and promote its degradation, then suppressed the activation of MAPK signaling pathway, hence ameliorated NASH in mouse (51). Besides, *LAPTM5* has been shown to be significantly upregulated in HCC tissues compared to normal liver tissues, and Pan et al. reported that *LAPTM5* could remarkably accelerate autophagic flux by promoting fusion of lysosomes with autophagosomes to drive lenvatinib resistance in HCC (52). Moreover, C3AR1 is a G protein-coupled receptor (GPCR) protein, which participates in the complement system and can stimulate the production of IL-1β and TGFβ (53). Interestingly, Han et al. found that *C3ar1* knockout mice showed drastically less severe fibrosing steatohepatitis, concomitantly with reduced hepatic stellate cells (HSCs) activation when compared with the wildtype littermates (54). In addition, the mRNA level of *LCP1* in liver tissue of NAFLD patients was strongly increased (300%) compare to the control group in a previous GWAS study (55), and Miller et al. used proteomic method to describe the proteome of NAFLD and observed that *LCP1* performed well in distinguishing the disease state from control group, NAFL from NASH and fibrosis grading (56). Notably, our study also found that the Turquoise module including hub gene *HLA-DRA*, displayed higher expression in NASH, which associated with NAFLD loci found by GWAS, and genetic variants of *HLA−DRA* has been recently reported to affect hepatitis development in a Korean population (57). Additionally, it has been shown that *SRGN*, *CD53*, *NCKAP1L*, *LCP1*, *EVI2B*, *MPEG1* and *TYROBP* may be potential pathological target gene for NAFLD and NASH, which is highly similar to our Turquoise module (58).

It should be noted that NASH is regarded as an inflammatory subtype of NAFLD with steatosis and evidence of hepatocyte injury and interactions between multiple immune cells. Increasing evidence has demonstrated the high heterogeneity and plasticity of macrophage populations in human liver (59). For example, Ramachandran et al. adopted scRNA-seq approach to discover a disease-associated TREM2+/CD9+ macrophage population that was remarkably expanded in human cirrhotic livers. Therapeutic inhibition of CCR2^+^ bone marrow-derived macrophages has been reported to alleviate inflammation and fibrosis in mouse NASH and fibrosis in human disease (36, 60). Similarly, our integrated scRNA-seq analysis revealed that the hub genes in the Turquoise module were mainly enriched in macrophage and dendritic cells, conforming the importance of which during NASH progression. For instance, our study found that expression of *FGL2* was elevated in macrophages and dendritic cells (**Figure 8C**). A recent study demonstrated that *Fgl2* expression in the livers of both humans and mice with NASH was significantly increased along with the accumulation of hepatic macrophages (61). Moreover, we found that the expression of *CSF1R* gene, a marker for pan-macrophages reported to be involved in hepatic fibrosis, was also considered as a potential marker for hepatocarcinogenesis (62). By analyzing the association between *LCP1* and immune cells, Zhang et al. found *LCP1* was significantly positively related to memory B cells as well as M1 macrophages (58). Our study also observed that hub gene *HLA-DRA* was higher expressed in both macrophages and dendritic cells (**Figure 8C**). Intriguingly, previous reports examining human NASH livers using single-cell RNA sequencing reported that M-Mac-1 included three genes, *HLA-DRA*, *HLA-DQA2* and *HLA-DQB2* (63), which was related to NAFLD loci (57, 64, 65). Further, recent study reported that cDC-related gene expression signatures in human livers were associated with NASH pathology (66). These findings emphasized the importance of further studies of the subpopulations of inflammatory macrophages and dendritic cells in NASH progression. However, more single-cell transcriptome data focusing on NASH progression among NASH patients are needed in future studies.

Several studies involving transcription factors have indicated therapeutic effects in NASH (67, 68), for example, transcription factors including *PPARs*, *LXR* and *FXR* are mainly known for their roles in altering lipid metabolism in NAFLD/NASH development. Agonists of *PPARs* and *FXR* have been investigated extensively in mouse models (69, 70), clinical trials presently are ongoing to test the effects of these drugs for potential NASH treatments. In addition, *PPARs*, *LXR* and *FXR* not only regulate lipid metabolism but also exert anti-inflammatory functions *via* direct and indirect mechanisms as shown by the suppression of several proinflammatory genes (71–74). Therefore, the detection of an immune-related transcription factor seems to be essential for the identification of novel therapeutic targets in NAFLD/NASH. In present study, we observed that the immune-related module enriched TFs including *NFKB1*, *STAT3*, *RFX5*, *ILF3*, *ELF1*, *SPI1*, *ETS1* and *CEBPA*, the expression of which enhanced with NASH progression (**Figure 9B**). Among the TFs, *NFKB1*, *STAT3*, *SPI1*, *ETS1*, *CEBPA* and *ELF1* have been reported to be linked to NAFLD/NASH by literature searching.

*NF-κB* is a protein complex that plays a central role in regulating the expression of cytokines and chemokines, and recent studies suggest that NF-κB is highly activated both in mice and patients with NASH (75, 76). *NFKB1* (p105/p50), a member of NF-κB family, emerging evidence suggests that NF-κB1-gene-coded proteins p105 and p50 have critical regulatory activities of inflammatory responses (77, 78). Previous study have showed that *Nfkb1*-deficient mice enhanced NASH progression to fibrosis by favouring NKT cell recruitment (79). In addition, Jurk et al. reported that loss of *Nfkb1* in mouse promoted ageing-related chronic liver disease, featured by steatosis, hepatitis, fibrosis and HCC (80), which point to the possible relevance of polymorphisms in human *NFKB1* gene as a risk factor for the progression of inflammatory disease (81).

*STAT* family members with inflammatory biological functions notably *STAT1* and *STAT3* have been linked to NAFLD and NASH. Grohmann and colleagues demonstrated that the oxidative hepatic environment in obesity restrained the *STAT1* and *STAT3* phosphatase *TCPTP*, which led to potentiate *STAT1* and *STAT3* signaling, and further increase the risk of developing NASH and HCC in the setting of nutritional excess (82). On the other hand, the suppression of *TCPTP*, coupled with heightened *STAT1* and *STAT3* signaling, were easily detectable events in the livers of patients with NASH (82). Moreover, a recently study revealed that dampening *IL6/STAT3* activity alleviated the I148M-mediated susceptibility to NAFLD, while boosting it in wild-type liver cultures enhanced the development of NAFLD (83). Additionally, downregulation of *STAT3* expression can activate autophagy and inhibit the inflammatory response of NASH (84, 85). Interestingly, other transcription factor such as *SPI1*, *ETS1* and *CEBPA* have been described to be a promising target for NASH prevention and treatment. Liu et al. applied proteomics strategy to identify *SPI1* as critical TF, *SPI1* expression was positively related to resistance indicator HOMA-IR and the inflammatory marker TNFA in human liver biopsies, and inhibition of *SPI1* ameliorated metabolic dysfunction and NASH (86). It has been proven that *Ets1* acted as a positive regulator of TGF-β1 signaling, which accelerated the development of NASH in mice (87). Notably, Vujkovic et al. recently presented a GWAS study and identified 77 genome-wide loci significantly associated with NAFLD (diagnosed using elevated ALT as a proxy for NAFLD), of interest is that for nine SNPs, the cATL risk allele was associated with lower BMI including *CEBPA* (65).

There are few studies of *RFX5*, *ELF1* and *ILF3* that have been reported at present in the field of NAFLD and NASH. *RFX5*, a classical transcription regulator of MHCII gene expression in the immune system. It has been previously shown that *RFX5* displayed higher transcriptional activity in both human NASH and mouse model of NASH (68). Interestingly, *RFX5* mRNA has previously been shown overexpressed in HCC compared with non-tumor tissue, which promoted HCC progression *via* transcriptionally activating *KDM4A*, *TPP1* and *YWHAQ* (88–90). Moreover, our results also showed that *RFX5* are the prominent regulators of expression of HLA class II genes in the immune-related module. Interestingly, *RFX5* was recently reported to enhance surface expression of *HLA-DR* molecules, which promoted tissue macrophages-dependent expansion of antigen-specific T cells in rheumatoid arthritis (91). In addition, *ELF1* regulated hub gene *CYBB* in MergeCohort_Turquoise module, the mechanism of TAZ-induced *Cybb* leading to liver tumor formation in NASH has been well defined (92).

*ILF3*, also known as NF90/NF110, encodes a double-stranded RNA (dsRNA)-binding protein which can regulate gene expression and stabilize mRNA (93, 94). Recent studies have reported insights into the possible physiological roles of *ILF3* in dyslipidemia, the cardiovascular system, neurodegenerative disorder as well as in tumorigenesis and progression of different cancers. Zhang et al. demonstrated that *ILF3* together with another eight transcription regulators control late-onset Alzheimer’s disease (LOAD) risk genes *HLA-DRB1* and *HLA-DQA1* expression in human microglial cells (95). Moreover, there is evidence that *ILF3* could have an important role in inflammatory pathophysiology *in vivo*, Nazitto et al. identified *ILF3* as negative regulator of innate immune response and dendritic cell (DC) maturation, and found that knockdown of *ILF3* led to significantly elevated expression of genes (*CD86*, *CD80* and *HLA-DR*) associated with DC maturation in the primary human monocyte-derived DCs during stimulation with viral mimetics or classic innate agonists (96). In addition, previous studies have revealed the essential roles of deregulated lncRNA *ILF3* divergent transcript (*ILF3-AS1*) in HCC, Bo et al. found that *ILF3-AS1* expression was significantly increased in HCC tissues and also associated with prognosis of HCC patients, and knockdown of ILF3-AS1 expression suppressed HCC cell proliferation, migration and invasion (97). Yan et al. also observed that *ILF3-AS1* silencing inhibited the hepatocellular carcinoma tumor growth (98). However, the regulation roles of *RFX5* and *ILF3* on *HLA-DR* molecules in the progression of NASH have also not been well defined. Therefore, our results provide a very meaningful direction for future research.

In summary, unlike previous studies with limitation of a few human NASH transcriptome data or focusing on individual genes influencing NASH progression, our network-driven strategy generated a comprehensive and unbiased view of the modules, hub genes and critical transcriptional factors associated with NASH. In particular, the Turquoise module and regulators involving immune-related pathways especially transcription factor *RFX5* coordinating antigen processing and presenting function in NASH progression deserve further attention. The main limitation of present study is that all conclusions are based on transcriptomic data from human and lack verification from relevant experiments *in vitro*/*in vivo* disease models. Nevertheless, it provides useful and novel molecular candidates in dysregulated pathways for NASH prognosis and therapeutic targets.

## Data availability statement

The original contributions presented in the study are included in the article/**Supplementary Material**. Further inquiries can be directed to the corresponding authors.

## Author contributions

Conception and design: J-JZ and FX. Acquisition and analysis of data: J-JZ, YS and X-YC. Investigation: J-JZ, YS, X-YC, M-LJ, F-HY and JZ. Software: J-JZ. Validation: YS, X-YC, M-LJ, S-LX and JZ. Visualization: J-JZ. Writing–original draft: J-JZ. Writing–review & editing: J-JZ, X-YC, F-HY and FX. Funding: J-JZ. All authors contributed to the article and approved the submitted version.

## Supplementary material

Supplementary Figure 1 Principal component analysis (PCA) of gene expression data set with the first two components. **(A)** PCA plot without batch effect elimination. **(B)** PCA plot with batch effect elimination with ComBat algorithm. PC1, first principal component; PC2, second principal component.

Supplementary Figure 2 Integrated scRNA-seq analysis. **(A)** Significant principal components (PCs) were determined via the JackStraw function in Seurat R-packages. PCs 1-17 were used for graph-based clustering (resolution = 0.4) to identify distinct clusters. **(B)** UMAP visualization of scRNA-seq data from four healthy (n = 2) and cirrhotic (n = 2) human livers annotated by liver sample. **(C)** UMAP visualization of cirrhotic and healthy control groups annotated by liver disease status. UMAP, uniform manifold approximation and projection.

Supplementary Figure 3 UMAP plots show the expression and distribution of top 25 hub genes in MergeCohort_Turquoise module for each cell type. The expression levels of those hub genes are expressed by the color transition from red to grey.

Supplementary Figure 4 Assessment of the expression patterns of hub genes and key TFs in MergeCohort_Turquoise module in vitro models of NASH using publicly available RNA-seq data of L02 hepatocytes (PRJNA726826) and murine primary hepatocytes (PRJNA726846) treated with palmitic acid and oleic acid (PAOA) for 0h, 12h and 24h, respectively. Heatmap shows the expression patterns of hub genes and key TFs in in L02 hepatocytes **(A)** and mouse primary hepatocytes **(B)** with PAOA treatment for 12 h and 24 h (1 technical replicate of 3 biological replicates for each group).

Supplementary Table 1 Characteristics of six liver transcriptome datasets from GEO comparing NASH patients with healthy controls (HC).

Supplementary Table 2 Significant enriched pathway of GSEA in Control-NASH group of five GEO datasets.

Supplementary Table 3 Significant enriched pathway of GSEA within the intersection of more than 4 list in Control-NASH group of five GEO datasets.

Supplementary Table 4 DEGs identified in MergeCohort between HC and NASH.

Supplementary Table 5 GO analysis of DEGs between HC and NASH.

Supplementary Table 6 Reactome pathways analysis of DEGs between HC and NASH.

Supplementary Table 7 Characteristics of gene modules obtained by WGCNA.

Supplementary Table 8 GO analysis of genes in each module.

Supplementary Table 9 KEGG pathways analysis of genes in each module.

Supplementary Table 10 DisGeNET enrichment analysis of genes in MergeCohort_Turquoise and GSE135251_Turquoise.

Supplementary Table 11 KEGG pathway analysis of genes in MergeCohort_Turquoise and GSE135251_Turquoise.

Supplementary Table 12 Transcription factor enrichment analysis by ChEA3 of MergeCohort_Turquoise module genes.
