# Supplementary figures and images for: Integrative network-based analysis on multiple Gene Expression Omnibus datasets identifies novel immune molecular markers implicated in non-alcoholic steatohepatitis
Source: Front Endocrinol (Lausanne). 2023 Mar 16;14:1115890. doi: 10.3389/fendo.2023.1115890 (PMC10061151; doi:10.3389/fendo.2023.1115890)

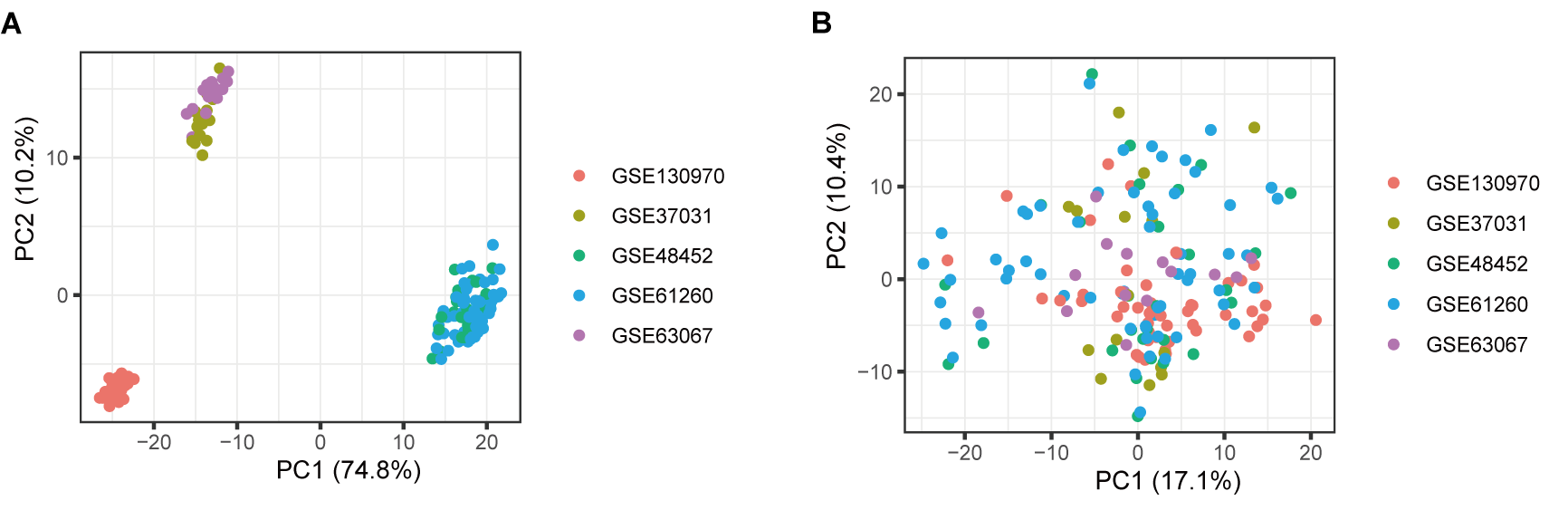

Supplement: Supplementary Figure 1 — Principal component analysis (PCA) of gene expression data set with the first two components. (A) PCA plot without batch effect elimination. (B) PCA plot with batch effect elimination with ComBat algorithm. PC1, first principal component; PC2, second principal component. [file DataSheet_1.zip › Image 1.TIF]

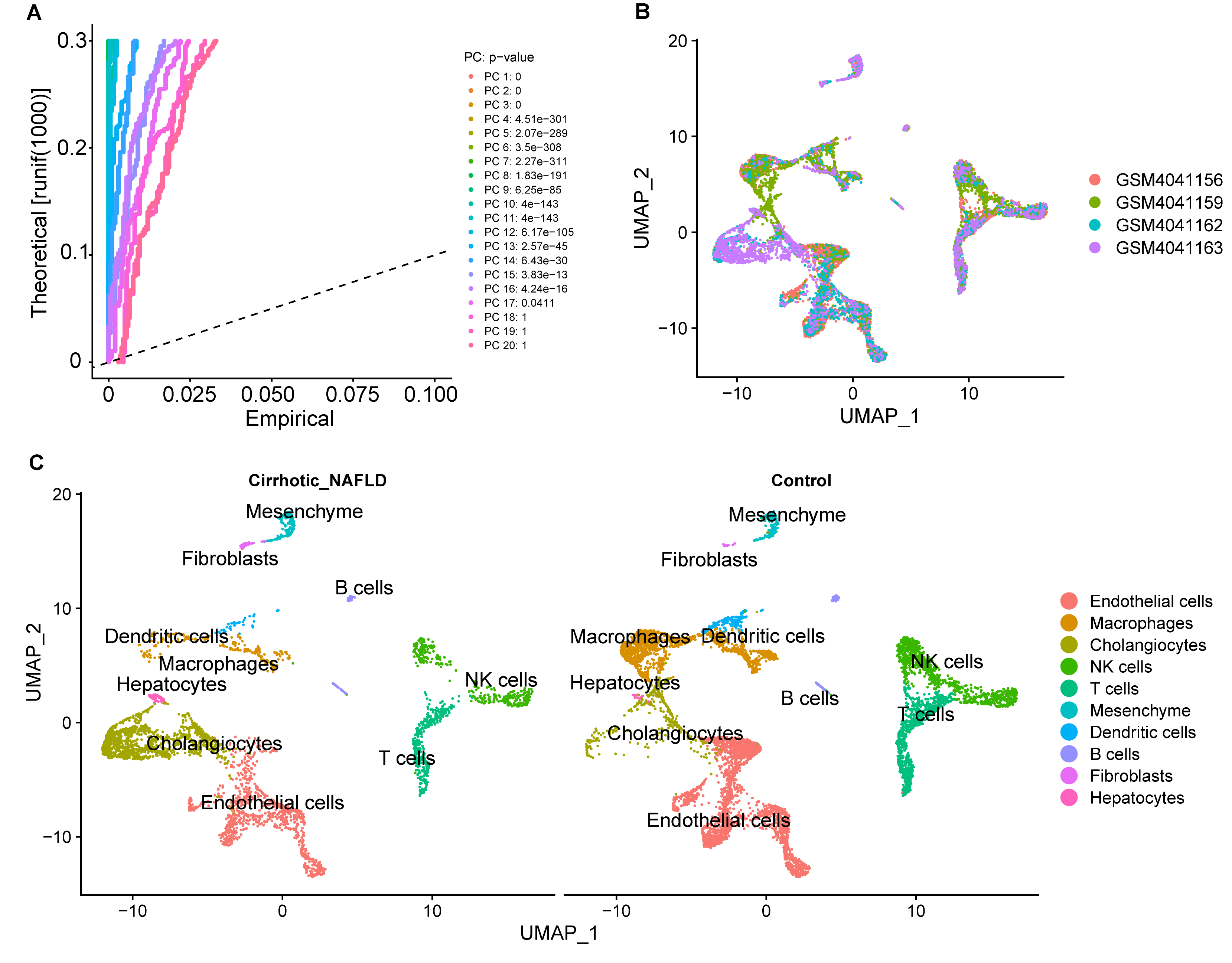

Supplement: Supplementary Figure 1 — Principal component analysis (PCA) of gene expression data set with the first two components. (A) PCA plot without batch effect elimination. (B) PCA plot with batch effect elimination with ComBat algorithm. PC1, first principal component; PC2, second principal component. [file DataSheet_1.zip › Image 2.TIF]

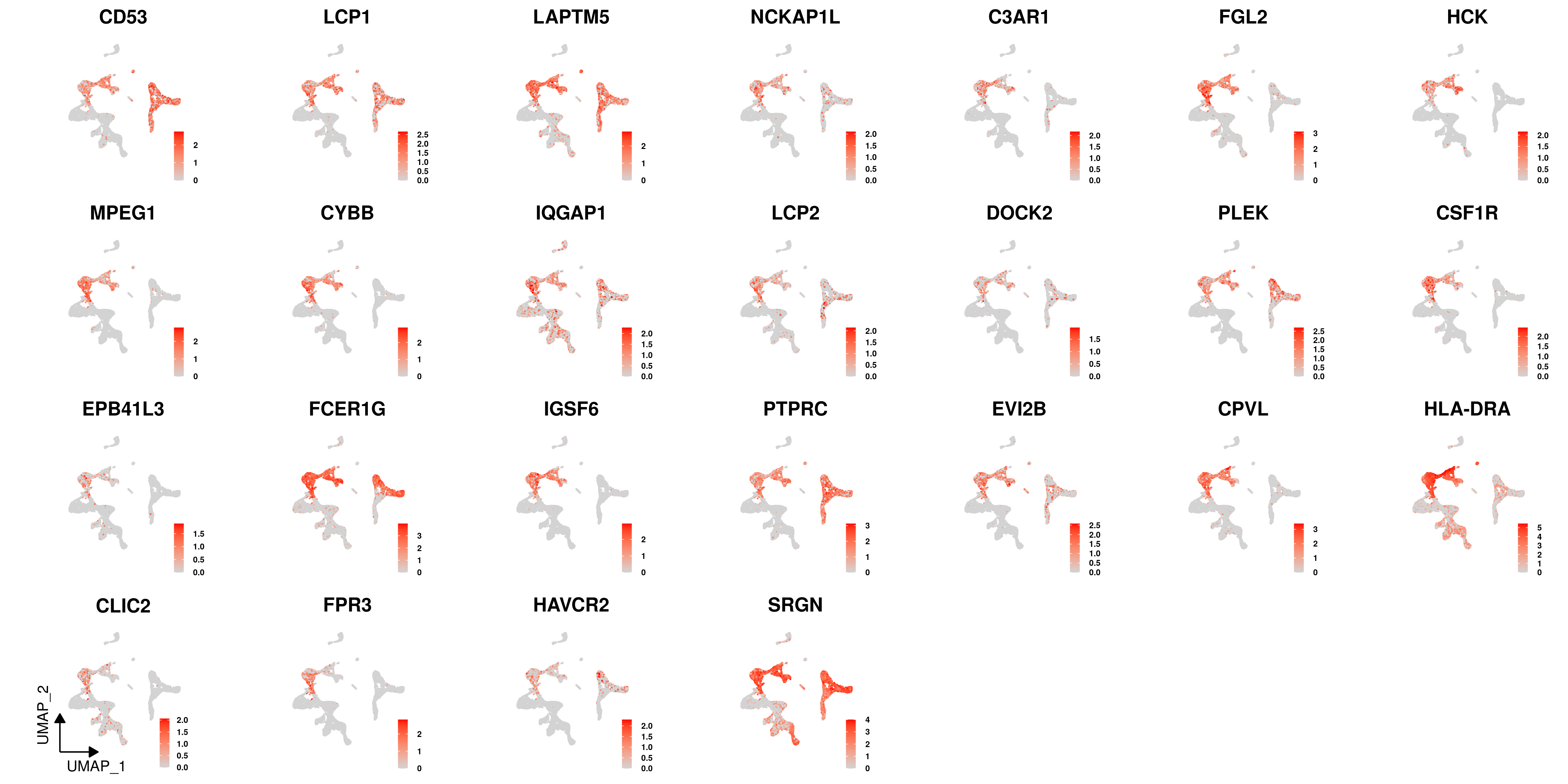

Supplement: Supplementary Figure 1 — Principal component analysis (PCA) of gene expression data set with the first two components. (A) PCA plot without batch effect elimination. (B) PCA plot with batch effect elimination with ComBat algorithm. PC1, first principal component; PC2, second principal component. [file DataSheet_1.zip › Image 3.TIFF]

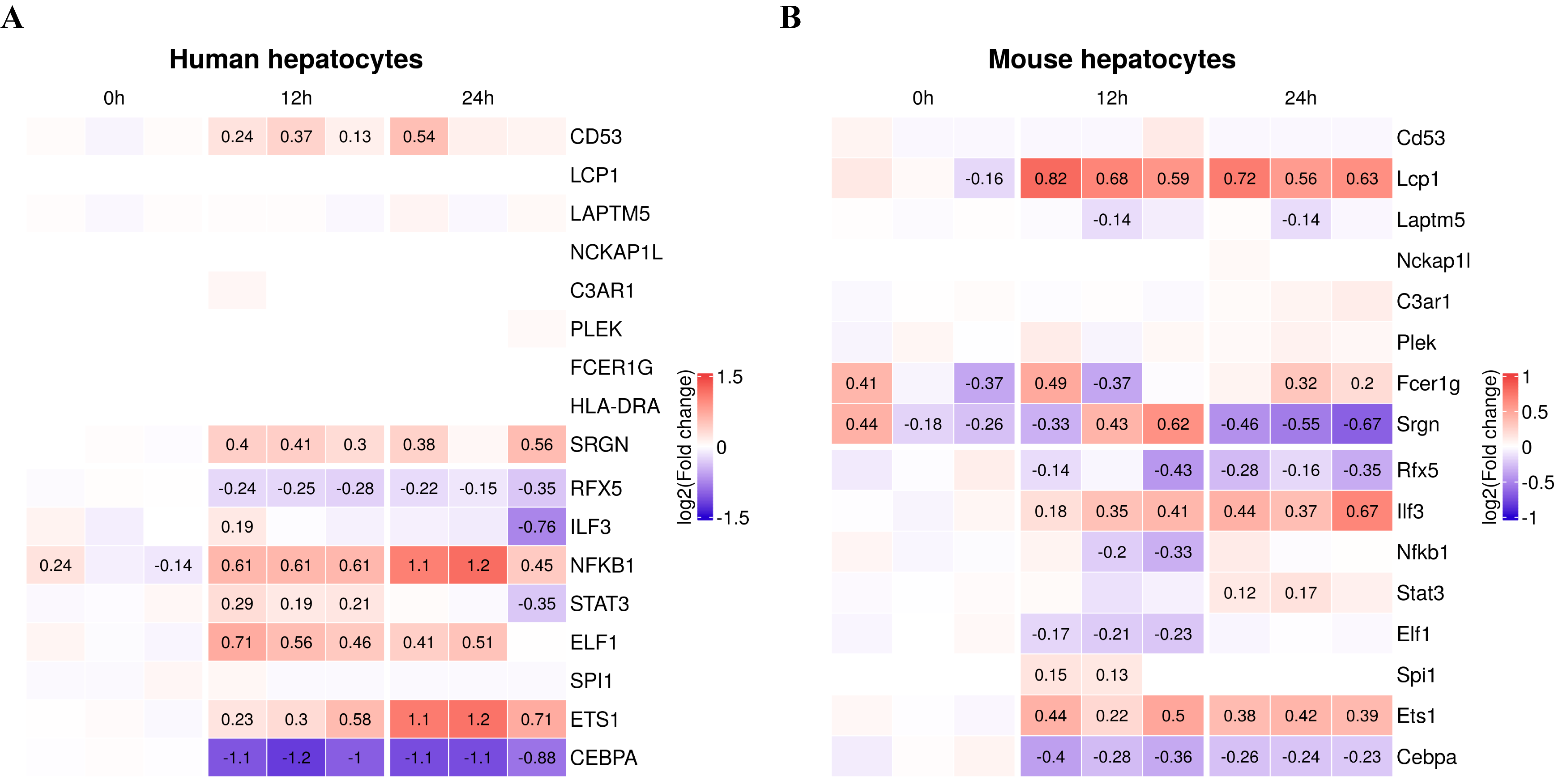

Supplement: Supplementary Figure 1 — Principal component analysis (PCA) of gene expression data set with the first two components. (A) PCA plot without batch effect elimination. (B) PCA plot with batch effect elimination with ComBat algorithm. PC1, first principal component; PC2, second principal component. [file DataSheet_1.zip › Image 4.TIF]
